# A Systematic Review and Meta-Analysis of Clinical Effectiveness and Safety of Hydrogel Dressings in the Management of Skin Wounds

**DOI:** 10.3389/fbioe.2019.00342

**Published:** 2019-11-21

**Authors:** Lijun Zhang, Hanxiao Yin, Xun Lei, Johnson N. Y. Lau, Mingzhou Yuan, Xiaoyan Wang, Fangyingnan Zhang, Fei Zhou, Shaohai Qi, Bin Shu, Jun Wu

**Affiliations:** ^1^Department of Burns, The First Affiliated Hospital, Sun Yat-sen University, Guangzhou, China; ^2^School of Public Health and Management, Chongqing Medical University, Chongqing, China; ^3^University of Hong Kong, Hong Kong Polytechnic University, Kowloon, China

**Keywords:** hydrogel, wound dressing, wound healing, pain relief, meta-analysis, systematic review

## Abstract

The purpose of this systematic review and meta-analysis is to assess the clinical effectiveness and safety of the medical hydrogel dressings used in skin wounds and therefore to weight the evidence for their clinical application. PubMed/Medline (1980–2019), Cochrane Library (1980–2019), ClinicalTrials.gov, Cochrane CENTRAL, Chinese Journal Full-text Database (CNKI, 1994–2019), and China Biomedy Medicine disc (CBM, 1978–2019), Chinese Scientific Journal Database (VIP, 1989–2019), and Wanfang Database (WFDATA, 1980–2019) were searched to identify relevant clinical trials and studies. Forty-three studies that assessed hydrogel vs. non-hydrogel dressings were identified. Compared to the latter, hydrogel dressings associated with a significantly shortened healing time of degree II burn (superficial and deep) wounds, diabetic foot ulcers, traumatic skin injuries, radioactive skin injuries, dog bites, and body surface ulcers. In addition, hydrogel dressing obviously increased the cure rate of diabetic foot ulcers, surgical wounds, dog bites, and body surface ulcers. Moreover, hydrogel dressing significantly relieved pain in degree II burn (superficial and deep) wounds, traumatic skin injuries, and laser treatment-induced wounds. However, no significant differences obtained between hydrogel and non-hydrogel dressings in the healing time of surgical wounds, the cure rate of inpatients' pressure ulcers, and phlebitis ulcers. This comprehensive systematic review and meta-analysis of the available evidence reveals that the application of hydrogel dressings advances the healing of various wound types and effectively alleviates the pain with no severe adverse reactions. These results strongly indicate that hydrogel products are effective and safe in wound management.

## Introduction

Skin is the largest human organ as it reaches almost 10% of the total body mass (Grice et al., 2009) and acts as a key protective barrier against the outside environment. Normally, the human body heal skin injuries via a set of complex and interactive processes that include hemostasis, inflammation, proliferation, and remodeling. However, this healing process can be impaired by various local and systemic factors causing more severe complications and a lower quality of life (Nourian Dehkordi et al., 2019). Plenty of wound care products have been created and developed in the latest decades aimed at promoting wound healing and improving the life quality of the patients afflicted by skin wounds (Metcalfe and Ferguson, 2007; Gil et al., 2013; Chattopadhyay and Raines, 2014; Garg et al., 2015; Xu et al., 2015; Das and Baker, 2016). Therefore, surgeons must specifically select wound treatment products according to the factors impeding wounds healing.

Since the 1960s, wound dressing was considered to play a positive role in wound healing. Wound dressing could establish and maintain an environment apt for wound repair. Winter (1962) were the pioneers of this field by initiating the concept of functional active dressings. According to them, the ideal advanced wound dressing should provide and maintain a moist environment, adequate gaseous exchange, and thermal insulation in the absence of toxic contaminants; it should protect against secondary infections, induce tissue regeneration, relieve wound pain, and promote wound healing quality; finally, it should be elastic, non-antigenic, and allow to manage wound exudate (Purna and Babu, 2000). Considering all the just mentioned factors, hydrogel products have the capacity to act as promising candidates as wound dressings for applications in clinical settings (Qu et al., 2018).

In 1960, Wichterle and Lim prepared the first hydrogels by cross-linking 2-hydroxyethyl methacrylate, thus initiating the application and practice of hydrogels in the biomedical field (Wichterle and Lím, 1960). Hydrogels are extremely hydrophilic. Advanced hydrogel materials are environment-sensitive or stimuli-sensitive, as they start swelling under certain conditions and respond to definite stimuli (Qiu and Park, 2001). They can absorb exudate from the wound surface and promote fibroblast proliferation and cell migration and keratinization. In addition, hydrogels' dense meshes can prevent bacteria from invading the wound while effectively transporting bioactive molecules (such as antibacterial agents and drugs) to the wound surface (Mohan et al., 2007; Tsao et al., 2010; Schwartz et al., 2012; Mao et al., 2017). At the same time, the unique mechanical properties of hydrogels i.e., elasticity and flexibility, allow for their adaptation to different parts of the wound, making them suitable for both wound care and tissue engineering (Huang et al., 2015).

Being a novel category of wet dressings, hydrogel products have been gradually perfected in recent years. Their clinical application has become rather extensive, ranging from dry scab wounds to multiple treatments of skin ulcers, burn wounds, animal bites, bed sores, etc. (Sood et al., 2014). Medicinal hydrogel dressings are endowed with a three-dimensional (3D) crosslinked network structure, which contains three main components, a high-molecular weight compound, propylene glycol, and water. High-molecular weight compounds such as Carboxy Methyl Cellulose (CMC) can double the absorption of wound exudate and necrotic tissue fluid (Roy et al., 2010). Propylene glycol can kill bacteria and prevent bacterial proliferation. In turn, the water in hydrogel dressings can create a relatively moist environment that prevents the wound from drying up (Fan et al., 2014). Therefore, although necrotic tissues in the making go through a slow hydration, the hydrogel dressing ensures a strong absorption of wound exudate. Concurrently, it promotes the debridement of water-soluble materials and absorbs wound carrion to provide a localized moist environment advancing wound healing (Qu et al., 2018). Besides, hydrogels' micro-acidic and hypoxic environment can attract cells involved in wound repair, help inhibit bacterial growth, and promote neoangiogenesis at the wound site (Dong et al., 2016).

Managing wounds through the use of hydrogels has been an accepted practice for decades. At present, many forms of hydrogel and non-hydrogel products are available aimed at managing wounds caused by various injuries. However, the benefits of multiple options also entail many challenges to the clinicians. The purpose of this systematic review and meta-analysis is to assess the clinical effectiveness and safety of the medicinal hydrogel dressings in treating multiple skin wounds compared to non-hydrogel dressings in terms of wound healing time, wound cure rate, pain reduction, and incidence of adverse reactions.

## Methods

### Systematic Review Eligibility Criteria

A systematic review was conducted according to the Preferred Reporting Items for Systematic Reviews and Meta-analyses (PRISMA) guidelines (Shamseer et al., 2015). It was based on the planned Participants, Intervention, Control, Outcome, and Study design (PICOS) elements outlined in Table 1.

**Table 1:** Inclusion and exclusion criteria.

**Criteria**	**Inclusion**	**Exclusion**
Type of study	RCTs, quasi-RCTs, CCTs	Review, case study, mechanism study, research and development, preparation and storage of materials, animal experiment, marketing strategy, editorials, news, and registered clinical trials with unfinished/unreported results.
Participants	Patients with skin wounds provoked by various causes (e.g., burns, surgery, body surface ulcers, etc.).	Patients with deep burns (degrees III and IV), treatment for bone wounds, pre-operation preparation, patients using biological tissue synthesis substitutes, and patients with autologous skin cultured transplants.
Interventions	Various types of hydrogel dressings [polymeric hydrophilic compounds such as guar gum and Lengningkang^a^ (Wound Caring)].	The hydrogel is used as a non-wound dressing such as an *in vivo* drug release carrier, contact lens, tissue filling material, medical sensor, etc.
Control	Any other dressing, treatment, placebo, or blank control.	Comparison of functions before and after using hydrogel dressings or comparison between different hydrogels.
Outcomes	Effective indicators including wound healing time, wound healing rate, pain score, pain level, etc. Safety indicators referring to the incidence rate of adverse reactions including skin allergy, skin dryness, tight skin, pruritus, and fever.	Long-term follow-up results such as quality of life.

a *The commercial name of a hydrogel dressing*.

### Search Strategy

We sought to identify suitable studies by searching the following online databases: PubMed/Medline (1980–2019), Cochrane Library (1980–2019), ClinicalTrials.gov, Cochrane CENTRAL, Chinese Journal Full-text Database (CNKI, 1994–2019), and China Biomedy Medicine disc (CBM, 1978–2019), Chinese Scientific Journal Database (VIP, 1989–2019), and Wanfang Database (WFDATA, 1980–2019). With the combination of subject words and free words, the search terms included two categories: (1) “hydrogel,” “polymeric hydrophilic compound,” “guar gum,” “guar bean,” and “polyvinylpyrrolidone (PVP);” (2) “wound,” “wound surface,” and “burn.” The logical relationship was created with “OR” and “AND,” and the search formula was thereafter developed according to the characteristics of the different databases. The search strategy was improved through a pre-retrieval process. Meanwhile, unpublished studies and conference materials were manually searched, and references of the included literature were also tracked. No language limits were applied.

### Study Selection

Two reviewers carried out the preliminary screening by independently reading titles and abstracts to exclude literature that obviously did not conform with the inclusion criteria. As a further screening they read the full texts of the literature that might meet the inclusion criteria. When the two researchers' opinions differed, they consulted and discussed with a third researcher to reach a final decision. During the full-text screening, the information below would be extracted: authors, date of publication, study type, subject characteristics, sample number, loss to or withdrawal from follow-up, intervention measures, and measuring indicators, etc. In case of multiple studies in a single published work, data based on study contents would be extracted as needed. With regard to repeatedly reported studies, only the latest or the most comprehensive one was included.

### Quality Evaluation

The quality of the methodology employed by the included studies was evaluated according to the Effective Practice and Organization of Care Group (EPOC) improved scoring standard recommended by The Cochrane Collaboration. The evaluation package included randomization methods, allocation concealment, blinding use, control of loss to follow-up, baseline information, outcome data, etc. Scores of 5–6 were classified as grade A, 2–4 as grade B, and 0–1 as grade C.

### Meta-Analysis

Meta-analysis was carried out by using the RevMan5.0 software recommended by The Cochrane Collaboration. Subgroups were divided according to patient (wound) types and types of outcome variables. The relative risk (RR) was taken as the combined effect size for categorical data, while the weighted mean difference (WMD) as the combined effect size for measuring data. Each effect size was shown as 95% CI. The heterogeneity of the study results was tested by χ^2^ test. When studies showed a statistical homogeneity (*P* > 0.1, *I*^2^ < 50%), a fixed-effect model would be used; otherwise, a random effect model was adopted. For subgroups containing a single study, description, and comparative analysis would be conducted on their results.

## Results

### Study Selection and Characteristics

**Figure 1:**
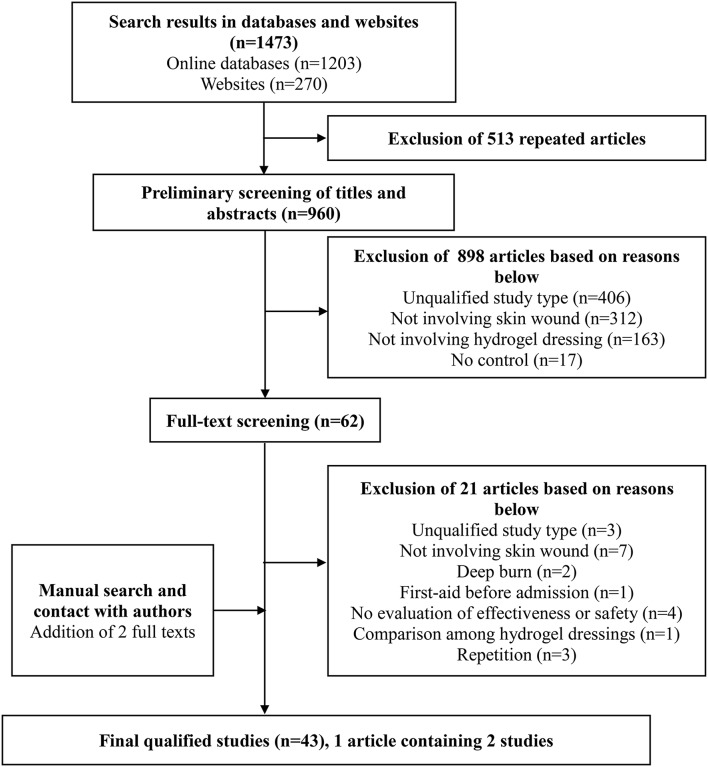
PRISMA flow diagram for inclusion or exclusion of studies used for systematic review.

One thousand four hundred and seventy three studies were selected by the preliminary screening. Only 43 studies were kept after screening titles, abstracts, and full-texts (Figure 1), including 29 randomized controlled trials (RCTs) and 14 clinical controlled trials (CCTs) with a total of 3,521 patients. The basic characteristics of the included studies and the results of methodological quality evaluations are shown in Table 2. In all studies, patients' basic situations were comparable between intervention groups and control groups (*P* > 0.05).

**Table 2:** Characteristics of the studies employing hydrogel dressings vs. non-hydrogel dressings.

**References**	**Study design**	**Country**	**Participants**	**Sample size**	**Quality level**
Cai et al., 2010	CCT	China	Degree-II deep burn wounds	60 patients Chitosan hydrogel = 30 SSD = 30	B
Jiang et al., 2008	RCT	China	degree-II superficial and deep burn wounds	90 patients Hydrogel = 45 SSD = 45	B
Wang et al., 2011	RCT	China	Degree-II superficial and deep burn wounds	560 patients Hydrogel with silver = 280 SSD = 280	B
Jin et al., 2009	CCT	China	Degree-II superficial and deep burn wounds	72 patients Hydrogel = 42 Iodine solution = 30	B
Wang et al., 2013	RCT	China	Degree-II burn wounds	76 patients Hydrogel = 38 Entoiodine and petrolatum gauze = 38	B
Liu, 2015	CCT	China	Degree-II superficial and deep burn wounds	120 patients Hydrogel and Lithosin solution = 60 Lithosin solution = 60	B
Jin et al., 2017	CCT	China	Degree-II superficial and deep burn wounds	92 patients Hydrogel = 48 SSD = 44	B
Diao et al., 2012	RCT	China	Degree-II superficial burn wounds	60 patients Hydrogel with silver = 30 SSD = 30	A
Lin et al., 2018	RCT	China	Degree-II superficial burn wounds	66 patients Hydrogel with silver = 33 SSD = 33	B
Liu and Ye, 2014	RCT	China	Degree-II superficial and deep burn wounds	80 patients Hydrogel = 40 Lithosin oil = 40	A
Shang, 2015	RCT	China	Degree-II deep burn wounds	68 patients Hydrogel = 34 Petrolatum gauze = 34	B
Li and Wu, 2018	CCT	China	Degree-II superficial and deep burn wounds	120 patients Hydrogel = 60 SD-Zn = 60	B
Lan and Duan, 2015	RCT	China	Degree-II deep burn wounds	60 patients Hydrogel with silver = 30 MEBO = 30	B
Gong et al., 2009	RCT	China	Degree-II superficial and deep burn wounds	104 patients Hydrogel with silver = 52 SSD and petrolatum gauze = 52	B
Cui et al., 2007	RCT	China	Degree-II superficial and deep burn wounds	44 patients Hydrogel = 22 SSD and Petrolatum gauze = 22	B
Xiang et al., 2012	CCT	China	Non-gangrenous diabetic foot ulcers	86 patients Alginate hydrogel with silver = 43 Polyvidone iodine = 43	B
Liu et al., 2017	RCT	China	Diabetic foot ulcers	30 patients Hydrogel = 15 Gentamicin dressing = 15	B
Teng, 2010	RCT	China	Diabetic foot ulcers	43 patients Hydrogel with silver = 23 Petrolatum gauze = 20	B
Shao et al., 2015	CCT	China	Diabetic foot ulcers	78 patients Hydrogel = 39 Glauber and Lidocaine hydrochloride = 39	B
Li et al., 2015	CCT	China	Diabetic foot ulcers	40 patients Hydrogel = 20 Iodophor oil and gauze = 20	B
Nie et al., 2015	RCT	China	Diabetic foot ulcers	65 patients Hydrogel with silver = 34 Petrolatum gauze = 31	B
Wang et al., 2008	RCT	China	Diabetic foot ulcers	43 patients Hydrogel with silver = 23 Petrolatum gauze = 20	A
Mao, 2010	RCT	China	Diabetic foot ulcers	44 patients Hydrogel with silver = 22 Silver dressing = 22	B
Zhang et al., 2012	RCT	China	Diabetic foot ulcers	126 patients Hydrogel with silver = 63 Silver dressing = 63	B
Chen et al., 2015	CCT	China	Diabetic foot ulcers	66 patients Hydrogel with silver = 33 Saline and petrolatum gauze = 33	B
D'Hemecourt et al., 1998	RCT	USA	Diabetic foot ulcers	138 patients Hydrogel = 70 Non-hydrogel = 68	A
Jensen et al., 1998	RCT	USA	Diabetic foot ulcers	31 patients Hydrogel = 14 Non-hydrogel = 17	B
Vandeputte and Gryson, 1997	RCT	Belgium	Diabetic foot ulcers	31 patients Hydrogel = 14 Non-hydrogel = 17	B
Huang et al., 2017	CCT	China	Pressure ulcers	45 patients Hydrogel = 23 Iodine and gauze = 22	B
Wen, 2015	RCT	China	Pressure ulcers	40 patients Hydrogel = 20 Betadine ointment = 20	B
Jiang et al., 2018	RCT	China	Radioactive skin injuries	108 patients Hydrogel = 54 Gauze = 54	B
Hu et al., 2015	RCT	China	Radioactive skin injuries	76 patients Hydrogel = 32 Gauze = 44	B
Shi et al., 2016	CCT	China	Phlebitis patients	73 patients Hydrogel = 38 Magnesium sulfate solution = 35	B
He et al., 2008	RCT	China	Phlebitis patients	60 patients Hydrogel = 30 Saline gauze = 30	B
Huang et al., 2016	RCT	China	Traumatic skin injuries	42 patients Hydrogel = 21 Multi-source therapy device = 21	B
Chen et al., 2015	CCT	China	Traumatic skin injuries	66 patients Hydrogel with silver = 35 Multi-source therapy device = 31	B
Zeng and Li, 2016	RCT	China	Traumatic skin injuries	44 patients Hydrogel = 22 Myogenic silicone = 22	A
Zeng and Li, 2016	RCT	China	Traumatic skin injuries	44 patients Hydrogel = 22 Myogenic cream and gauze = 22	A
Lu et al., 2017	CCT	China	Surgical wounds	62 patients Hydrogel with silver = 31 Gauze = 31	B
Fan et al., 2013	RCT	China	Surgical wounds	100 patients Hydrogel with silver = 42 Gauze = 58	A
Wang et al., 2008	RCT	China	Canine bites	40 patients Hydrogel with silver = 20 Saline and gauze = 20	A
Fang et al., 2011	CCT	China	Body surface ulcers	72 patients Hydrogel with silver = 36 Iodine, hydrogen peroxide, and petrolatum gauze = 36	B
Fan et al., 2014	RCT	China	Laser treatments	200 patients Hydrogel = 100 Non-hydrogel = 100	B

### Data Synthesis

#### Healing Times Comparison of Degree-II Superficial and Deep Burn Wounds

**Figure 2:**
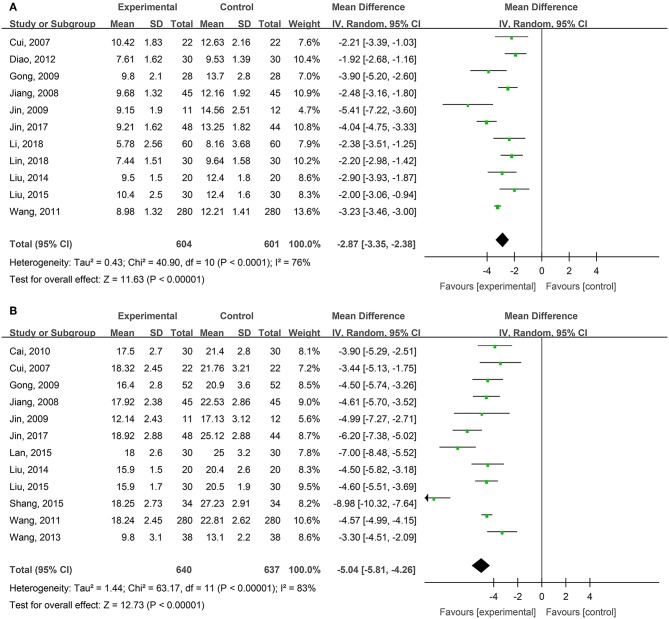
Comparative meta-analysis of the healing times of degree-II superficial **(A)** and degree-II deep **(B)** burn wounds.

Eleven studies, reported by Cui et al. (2007), Jiang et al. (2008), Gong et al. (2009), Jin et al. (2009), Wang et al. (2011), Diao et al. (2012), Liu and Ye (2014), Liu (2015), Jin et al. (2017), Li and Wu (2018); Lin et al. (2018), compared the healing times of degree-II superficial burn wounds treated with hydrogel dressings or other treatments. There existed a statistical heterogeneity among the study results (*P* < 0.0001, *I*^2^ = 76%). Therefore, the random effect model was applied for meta-synthesis (Figure 2A). The results showed that on average the wound healing time of the hydrogel dressings group was shortened by 2.87 days as compared with the control group and that the difference had a high statistical significance (MD = −2.87, 95% CI: −3.35 to −2.38, *P* < 0.00001).

Twelve studies, reported by Cui et al. (2007), Jiang et al. (2008), Gong et al. (2009), Jin et al. (2009), Cai et al. (2010), Wang et al. (2011), Wang et al. (2013), Liu and Ye (2014), Lan and Duan (2015), Liu (2015), Shang (2015), and Jin et al. (2017), compared the healing times of degree-II deep burn wounds treated with hydrogel dressings or other therapeutics. There existed a statistical heterogeneity among the study results (*P* < 0.00001, *I*^2^ = 83%). Hence, the random effect model was applied for meta-synthesis (Figure 2B). The results revealed that on average the wound healing time of the hydrogel dressings group was shortened by 5.04 days as compared with the control group and that statistically this difference was highly significant (MD = −5.04, 95% CI: −5.81 to −4.26, *P* < 0.00001).

#### WHO Pain Ratings of Burn Wounds

**Figure 3:**
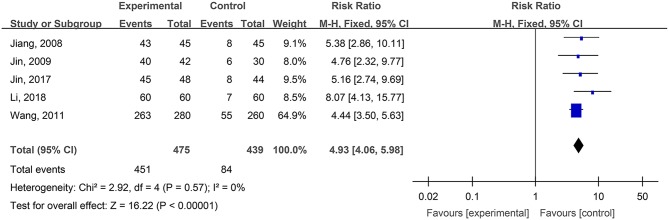
Comparative meta-analysis of WHO pain ratings of burn wounds.

Five studies, reported by Jiang et al. (2008), Jin et al. (2009), Wang et al. (2011), Jin et al. (2017), and Li and Wu (2018), compared the pain ratings difference of burn wounds after treatment with hydrogel dressings or other therapeutic means. There occurred no statistical heterogeneity among the study results (*P* = 0.57). Consequently, the fixed effect model was applied for meta-synthesis (Figure 3). The results brought to light that patients suffering either grade 0 or grade I pain accounted for a higher proportion among those treated with hydrogel dressings and that statistically the difference was highly significant (OR = 4.93, 95% CI: 4.06–5.98, *P* < 0.00001).

#### VAS Pain Scores of Degree-II Superficial and Deep Burn Wounds

**Figure 4:**
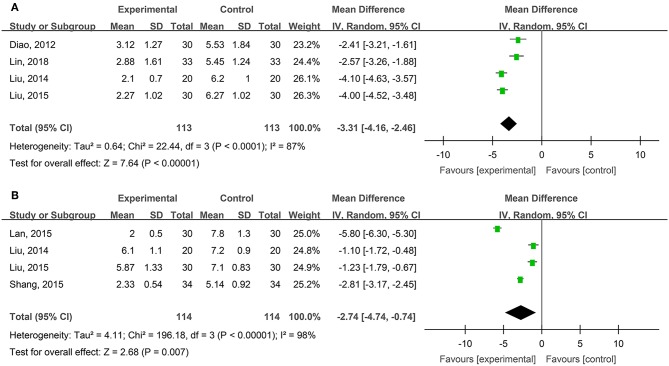
Comparative meta-analysis of VAS pain scores of degree-II superficial **(A)** and deep **(B)** burn wounds.

Four studies, reported by Diao et al. (2012), Liu and Ye (2014), Liu (2015), and Lin et al. (2018), compared visual analog scale (VAS) pain scores of the burn wounds treated with hydrogel dressings or other therapeutics. There occurred a statistical heterogeneity among the study results (*P* < 0.00001, *I*^2^ = 87%). Accordingly, the random effect model was applied for meta-synthesis (Figure 4A). The results showed that on average the VAS score of the hydrogel dressings group was 3.31 points lower than the control group, and that the difference had a high statistical significance (MD = −3.31, 95% CI: −4.16 to −2.46, *P* < 0.00001).

Four studies, reported by Liu and Ye (2014), Lan and Duan (2015), Liu (2015), and Shang (2015), compared VAS pain scores of burn wounds treated with hydrogel dressings or other medicaments. A statistical heterogeneity turned up among the study results (*P* < 0.00001, *I*^2^ = 98%). For that reason, the random effect model was applied for meta-synthesis (Figure 4B). The results made clear that on average the VAS score of the hydrogel dressings group was 2.74 points lower than that of the control group and that the difference was statistically significant (MD = −2.74, 95% CI: −4.74 ~ −0.74, *P* = 0.007).

#### Wound Healing Times of Diabetic Foot Ulcers

**Figure 5:**
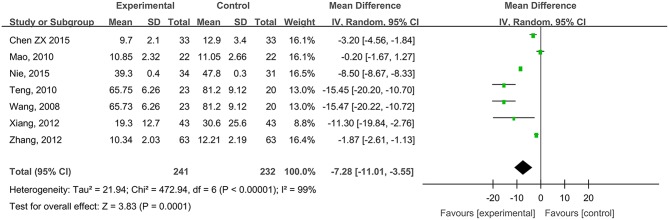
Comparative meta-analysis of wound healing times of diabetic foot ulcers.

Seven studies, reported by Wang et al. (2008), Mao (2010), Teng (2010), Xiang et al. (2012), Zhang et al. (2012), Chen (2015), and Nie et al. (2015), compared the healing times of diabetic foot ulcer wounds treated with hydrogel dressing or other ministrations. There occurred a statistical heterogeneity among the study results (*P* < 0.00001, *I*^2^ = 99%). Therefore, the random effect model was applied for meta-synthesis (Figure 5). The results made plain that on average the healing time of the hydrogel dressings group was 7.28 days shorter than that of the control group and that the difference had a high statistical significance (MD = −7.28, 95% CI: −11.01 to −3.55, *P* < 0.0001).

#### Wound Cure Rates of Diabetic Foot Ulcers

**Figure 6:**
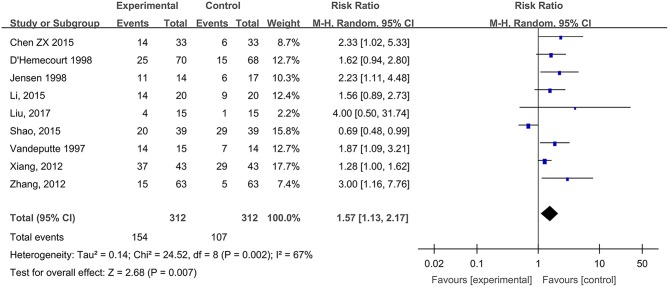
Comparative meta-analysis of wound cure rates of diabetic foot ulcers.

Nine studies, reported by Vandeputte and Gryson (1997), D'Hemecourt et al. (1998), Jensen et al. (1998), Xiang et al. (2012), Zhang et al. (2012), Chen (2015), Li et al. (2015), Shao et al. (2015), and Liu et al. (2017), compared the wound cure rates of diabetic foot ulcers treated with hydrogel dressing or other therapeutics. There existed a statistical heterogeneity among the study results (*P* = 0.002, *I*^2^ = 67%). Hence, the random effect model was applied for meta-synthesis (Figure 6). The results proved that the cure rate of diabetic foot ulcers was higher in the hydrogel dressings group than in the control group and that the difference was statistically significant (RR = 1.57, 95% CI: 1.13–2.17, *P* = 0.007).

#### Healing Times of Traumatic Skin Injuries

**Figure 7:**
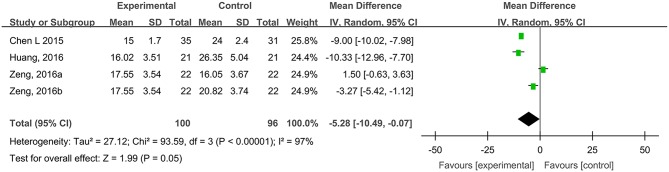
Comparative meta-analysis of healing times of traumatic skin injuries.

Four studies, reported by Chen et al. (2015), Huang et al. (2016), and Zeng and Li (2016), compared the healing times of traumatic skin injuries treated with hydrogel dressings or other therapeutics. There occurred a statistical heterogeneity among the study results (*P* < 0.00001, *I*^2^ = 97%). Consequently, the random effect model was applied for meta-synthesis (Figure 7). The results revealed that on average the healing time of traumatic skin injuries was 5.28 days shorter in the hydrogel dressing group than in the control group and that the difference reached statistical significance (MD = −5.28, 95% CI: −10.49 to −0.07, *P* = 0.05).

#### WHO Pain Ratings of Traumatic Skin Injuries

**Figure 8:**
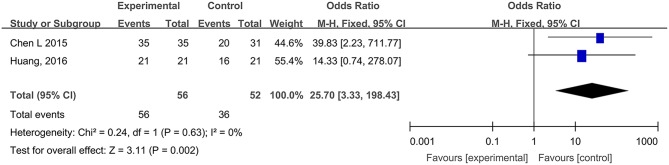
Comparative meta-analysis of WHO pain ratings of traumatic skin injuries.

Two studies, reported by Chen et al. (2015) and Huang et al. (2016), compared the WHO pain ratings difference after treatment with hydrogel dressings or other therapeutic interventions. There existed no statistical heterogeneity among the study results (*P* = 0.63). In consequence, the fixed effect model was applied for meta-synthesis (Figure 8). The results disclosed that patients suffering grade-0 and grade-I pain accounted for a higher proportion than the control group did and that the difference was statistically significant (RR = 25.70, 95% CI: 3.33–198.43, *P* = 0.002).

#### Healing Times and Cure Rates of Surgical Wounds

**Figure 9:**
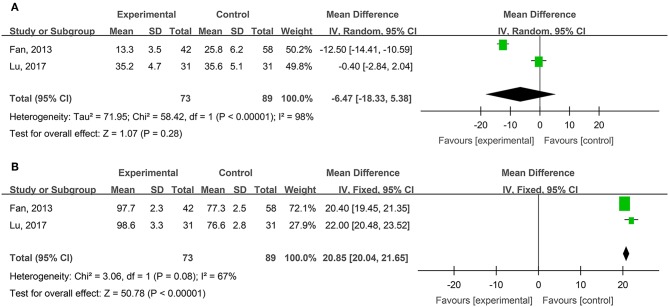
Comparative meta-analysis of healing times **(A)** and cure rates **(B)** of surgical wounds.

Two studies, reported by Fan et al. (2013) and Lu et al. (2017), compared the healing times of surgical wounds treated with hydrogel dressing or other ministrations. There existed a statistical heterogeneity among the study results (*P* < 0.00001, *I*^2^ = 98%). Therefore, the random effect model was applied for meta-synthesis (Figure 9A). The results showed that as the healing time of surgical wounds was concerned no statistically significant difference (*P* = 0.28) intervened between the hydrogel dressings group and the control group.

Two studies, reported by Fan et al. (2013) and Lu et al. (2017), compared the cure rates of surgical wounds medicated with hydrogel dressing or other treatments. There existed no statistical heterogeneity among the study results (*P* = 0.08). Consequently, the fixed effect model was applied for meta-synthesis (Figure 9B). The results demonstrated that the cure rate of surgical wounds in the hydrogel dressings group was 20.85% higher than in the control group and that statistically the difference was highly significant (MD = 20.85%, 95% CI: 20.04–21.65%, *P* < 0.00001).

#### The Cure Rates of Inpatients' Pressure Ulcers

**Figure 10:**
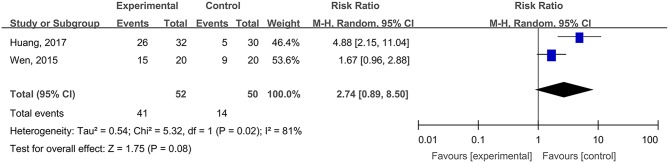
Comparative meta-analysis of cure rates of inpatients' pressure ulcers.

Two studies, reported by Wen (2015) and Huang et al. (2017), compared the cure rates of inpatients' pressure ulcers treated with hydrogel dressings or other therapeutic means. There existed a statistical heterogeneity among the study results (*P* = 0.002, *I*^2^ = 81%). Hence, the random effect model was applied for meta-synthesis (Figure 10). The results revealed that there occurred no statistically significant difference between the hydrogel dressing group and the control group (*P* = 0.08) in the cure rate of inpatients' pressure ulcers.

#### Healing Times of Radioactive Skin Injuries

**Figure 11:**
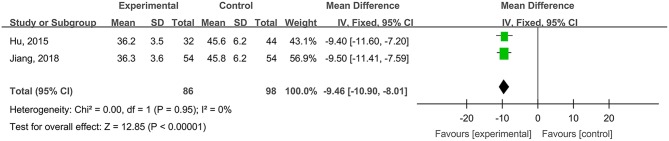
Comparative meta-analysis of healing times of radioactive skin injuries.

Two studies, reported by Hu et al. (2015) and Jiang et al. (2018), compared the healing times of radioactive skin injuries treated with hydrogel dressings or other medicaments. There occurred no statistical heterogeneity among the study results (*P* = 0.95). In consequence, the fixed effect model was applied for meta-synthesis (Figure 11). The results demonstrated that on average the healing time of the hydrogel dressings group was shortened by 9.46 days as compared with that of the control group and that the difference had a high statistical significance (MD = −9.46, 95% CI: −10.90 to −8.01, *P* < 0.00001).

#### The Cure Rates of Phlebitis Ulcers (Cure and Effectiveness)

**Figure 12:**
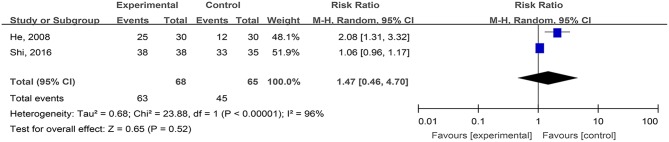
Comparative meta-analysis of cure rates of phlebitis ulcers.

Two studies, reported by He et al. (2008) and Shi et al. (2016), compared the cure rates of phlebitis ulcers treated with hydrogel dressings and other ministrations. There existed a statistical heterogeneity among the study results (*P* < 0.00001, *I*^2^ = 96%). Consequently, the random effect model was applied for meta-synthesis (Figure 12). The results indicated that the difference in cure rates between the hydrogel dressings group and the control group of patients with phlebitis ulcers was not statistically significant (*P* = 0.52).

#### Dog Bite Wounds, Body Surface Ulcers, and Laser Treatment-Induced Wounds

Only one study, reported by Wang and Teng (2008), compared the cure rates of dog bite wounds treated with hydrogel dressings or saline gauze. The results made known that the healing time of the hydrogel dressings group was 4.0 days shorter than that of controls (*t* = −16.54, *P* < 0.001); in addition, the average cure rate of the wounds was 24.8% higher (*t* = 27.8, *P* < 0.001) than the controls.

Then again, a single study, reported by Fang et al. (2011), compared the cure rates of body surface ulcers treated with hydrogel dressings or conventional therapy with Iodophor or hydrogen peroxide plus Vaseline gauze. The results revealed that the healing time of the hydrogel dressings group was 18.4 days shorter than that of the controls (*t* = −5.29, *P* < 0.001); moreover, the total wound cure rate was also significantly higher than that of the control group (χ^2^ = 13.78, *P* < 0.001).

Finally, a lone study, reported by Xin et al. (2014), compared the wound care of patients categorized as hydrogel dressings group and blank control group bearing laser treatment-induced wounds. Concerning VAS scores, as contrasted with the blank control group, the pain score of the hydrogel dressings group was 1.63 lower (*t* = −6.47, *P* < 0.001), the burning sensation score was 1.10 lower (*t* = −8.65, *P* < 0.001) and the stimulating sensation score was 1.46 lower (*t* = −10.78, *P* < 0.001) than the controls.

### Data Set of Complaints and Adverse Events

#### Data Source

Besides the mentioned above Chinese and English databases, a supplementary search was carried out in the State Monitoring System of Drug Adverse Reactions (SMSDAR; http://www.adrs.org.cn/).

#### Data Synthesis and Analysis

To perform Meta-analyses about the incidence rate of adverse reactions RevMan5.0 software was used and the relative risk was taken as a combined effect size. The heterogeneity of the study results was tested by χ^2^-test. When the study showed a statistical homogeneity (*P* > 0.1, *I*^2^ < 50%), a fixed effect model was applied, otherwise a random effect model was adopted.

#### Analysis Result

**Figure 13:**
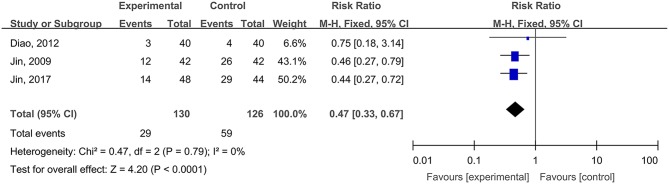
Comparative meta-analysis of the incidence rates of adverse reactions of burn wound-affected patients.

Three studies, reported by Jin et al. (2009), Diao et al. (2012), and Jin et al. (2017), compared the adverse reaction rates in cases of burn wounds treated with hydrogel dressings or other therapeutics. No statistical heterogeneity was detected among the study results (*P* = 0.79). Therefore, the random effect model was applied for meta-synthesis (Figure 13). The results disclosed that the incidence rate of adverse reactions—including skin dryness, swelling, pruritus, and fever—was lower in the hydrogel dressings group than in the control group, and that statistically the difference was highly significant (RR = 0.47, 95% CI: 0.33–0.67, *P* < 0.0001). Other included studies reported no details about patients' adverse reactions.

No reports on adverse reactions of using medicinal hydrogels were found in the State Monitoring System of Drug Adverse Reactions (SMSDAR).

## Discussion

This study attempted to adopt the Cochrane systematic evaluation and Meta-analysis to assess the effectiveness and safety of hydrogel dressings employed in the management of skin wounds. The results brought to light that the application of medicinal hydrogel dressings can significantly shorten the healing time of skin wounds such as superficial degree-II burns (Figure 2A), deep degree-II burns (Figure 2B), diabetic foot ulcers (Figure 5), traumatic skin injuries (Figure 7), radioactive skin injuries (Figure 11), dog bites (*t* = −5.29, *P* < 0.001), and body surface ulcers (*t* = −5.29, *P* < 0.001). Hydrogel dressings can also effectively improve the cure rate of diabetic foot ulcers (Figure 6), surgical wounds (Figure 9B), dog bites (*t* = 27.8, *P* < 0.001), and body surface ulcers (χ^2^ = 13.78, *P* < 0.001). These advantageous effects are likely due to the nearly ideal moist environment that hydrogel dressings provide once applied to skin wounds. This promotes cell viability and physiological functioning and subsequently wound healing. In addition, hydrogel dressings reduce the loss of body fluids while absorbing wound's exudate and advancing autolytic debridement in necrotic wounds and granulating wounds. The hydrogels' swelling property has been proved to decrease the excessive fluid accumulation between the wound surface and the dressing. On the other hand, the hydrogel owns a soft texture and tends to adhere to the wound surface tightly and evenly, which prevents bacterial invasion and reduces soreness as well.

In recent years, with the appearance of new antibiotics and drugs applied to wounds, bactericidal and bacteriostatic substances such as silver ions have been combined with dressings to control local infections and accelerate wound healing. Nanocrystalline silver modulates the inflammatory response through its antimicrobial activity, thereby reducing the infections incidence and leading to an improved wound healing outcome. Furthermore, a faster re-epithelialization occurred in the wounds treated with nanocrystalline silver-coupled dressing rather than with a standard antibiotic solution (Demling and DeSanti, 2002; Nherera et al., 2017).

Study results also indicate that medicinal hydrogels can effectively alleviate the pain and burning and irritating sensations typical of skin wounds. The WHO pain rating of burn wounds (Figure 3) and traumatic skin injuries was significantly lower in the hydrogel dressing treatment studies. In addition, when hydrogel dressings were compared with non-hydrogel treatments, the VAS pain score was obviously lower in superficial degree-II burns (Figure 4A), deep degree-II burns (Figure 4B), and laser treatment-induced wounds. Concurrently, adverse reactions such as wound dryness, swelling, pruritus, and fever were significantly reduced (Figure 13). The benefits brought by hydrogel dressings to wounds might be related to the hydrogel-induced microenvironment that minimizes secondary injuries and alleviates pain by generating a cool feeling and by protecting any exposed peripheral nerve terminals. Our data also indicated that the guar gum-based hydrogel (CQ-01) is safe and can effectively alleviate the intractable pruritus otherwise affecting the patients [the score of Jun Wu scale (JW scale) pruritus rating scale for CQ-01 group was significantly lower than that of the traditional dressing group]. This further supports the clinical antipruritic effect of hydrogel dressings (Wu et al., 2016).

Meta-analysis is an observational study, thus, biases are somehow inevitable (Easterbrook et al., 1991). Among 43 original studies only 8 of them were graded A according to EPOC quality grading, which may potentially prejudice the results. Moreover, some hydrogel dressings were used in combination with other dressings, for example, silver dressings or with Lithosin solution. None of these trials assessed the effects of these combinations. It should be noted that hydrogel dressings are supposed to be applied singly rather than in combination with other therapeutics and that when used in combination their effectiveness and safety cannot be evaluated from individual dressing data. On the other hand, in the result of healing times comparison of burn wounds and others, there existed a statistical heterogeneity among the study results. The main reason for statistical heterogeneity of selected studies is that the sample size of each selected study varies greatly. In addition, clinical heterogeneity may also cause heterogeneity in statistical analysis, such as differences in baseline characteristics and medical conditions of burn patients in various studies, which may affect treatment outcomes.

The limitation of this meta-analysis is that, various dressings were applied in control groups included in this review, such as SSD, Iodine solution, Entoiodine and petrolatum gauze, Lithosin solution and oil, SD-Zn, Petrolatum gauze, Polyvidone iodine, Gentamicin dressing, etc. which may affect the outcomes and potentially add the biases to the study as well.

The main limitation of this review is the potential publication bias in terms of safety assessment of hydrogel dressings. Although we endeavored to collect quite a number of clinical trials by searching both publication databases and SMSDAR, in this systematic review only three studies compared the adverse reactions between hydrogel dressings and other medicinal products. The poor reporting of adverse reactions could be generalizable to the study purpose of clinical trials, which are commonly designed to explore the effectiveness of a dressing in promoting wound healing while they do not focus on the wound site responses to the dressing tested. On the other hand, it is sometimes hard to distinguish an adverse reaction from events related to wound healing.

## Conclusions

This evidence-based systematic review and meta-analysis from RCTs and CCTs studies suggests that the use of hydrogel dressings results in a significant decrease in wound healing time, an obvious increase in cure rate, and a satisfying relief of pain as compared to non-hydrogel dressings. All the above-reported results strongly indicate that hydrogel products are effective and safe in wound management. Furthermore, there is a need for high-quality and international multi-center RCTs reporting adverse reactions to help clinicians make informed decisions on the best options for patients suffering from skin wounds.

## Data Availability Statement

All datasets generated for this study are included in the article/supplementary material.

## Author Contributions

Study design and conception of this manuscript were due to JW and BS. Literature retrieving and studies selection were performed by XL and JL. MY and XW carried out the quality evaluation of the study. Mathematical modeling and meta-analysis were conducted by FZha and FZho. Results analysis and interpretation were done by LZ, HY, and SQ. The manuscript was drafted by LZ and HY. All authors read and approved the final manuscript.

### Conflict of Interest

The authors declare that the research was conducted in the absence of any commercial or financial relationships that could be construed as a potential conflict of interest.

## Abbreviations

GRADE: Grading of Recommendations Assessment, Development and Evaluation
PRISMA: Preferred Reporting Items for Systematic Reviews and Meta-Analyses
RCT: Randomized Controlled Trial
PICOS, Participants, Intervention, Control: Outcome and Study design
PVP: Polyvinylpyrrolidone
CMC: Carboxy Methyl Cellulose
EPOC: Effective Practice and Organization of Care Group
WMD: Weighted Mean Difference
SSD: Silver Sulphadiazine
VAS: Visual Analog Scale
SMSDAR: State Monitoring System of Drug Adverse Reactions
JW scale: Jun Wu scale.
